# Intralesional corticosteroid injections in the treatment of central giant cell lesions of the jaws: A meta-analytic study

**DOI:** 10.4317/medoral.18345

**Published:** 2013-02-05

**Authors:** Rafael L. Osterne, Phelype M. Araújo, Abrahao C. de Souza-Carvalho, Roberta B. Cavalcante, Eduardo Sant’Ana, Renato L. Nongueira

**Affiliations:** 1 DDS, MSc, Assistant Professor, Department of Pathology, Fortaleza University School of Medicine, Fortaleza, Brazil; 2 DDS, MSc student, Federal University of Ceará School of Dentistry, Fortaleza, Brazil; 3 DDS, MSc, PhD, Assistant Professor, Faculdade Católica Rainha do Sertão, School of Dentistry, Quixadá, Brazil; 4 DDS, MSc, PhD, Associate Professor, Department of Oral and Maxillofacial Pathology, Fortaleza University School of Dentistry, Fortaleza, Brazil; 5 DDS, MSc, PhD, Associate Professor of Oral and Maxillofacial Surgery, Bauru School of Dentistry, University of São Paulo, Bauru, Brazil; 6 DDS, MSc, PhD, Associate Professor, Department of Dental Clinic, Discipline of Oral and Maxillofacial Surgery and Stomatology, Federal University of Ceara School of Dentistry, Fortaleza, Brazil. Oral and Maxillofacial Surgeon, Department of Oral and Maxillofacial Surgery, Memorial Batista Hospital, Fortaleza, Brazil

## Abstract

Objective: The aim of this study was to evaluate the response of treatment of central giant cell lesion to intralesional corticosteroid injections. Study Design: Review of articles indexed in PubMed on the topic between the years 1988 and 2011, and development of a descriptive meta-analysis of the results. Results: Sample of 41 patients primarily treated with intralesional corticosteroid injections was obtained, with a male female ratio of 1:0.95, being 23 aggressive and 18 non-aggressive central giant cell lesions. Triamcinolone acetonide and triamcinolone hexacetonide were the drugs used, and 78.0% cases were considered as good result, 14.6% were considered as moderate response and 7.3% were considered as negative result to treatment. Considering the aggressiveness, 88.9% of non-aggressive lesions presented a good response to treatment, in aggressive central giant cell lesions, 69.6% presented a good response to intralesional corticosteroid injections. Conclusion: In view of the results analyzed, intralesional corticosteroid injections could be considered as first treatment option for central giant cell lesion.

** Key words:**Central giant cell lesion, corticosteroids injections, triamcinolone hexacetonide, triamcinolone acetonide.

## Introduction

Central giant cell lesion (CGCL) is an uncommon type of benign jaw lesion that can be classified as aggressive or non-aggressive (1,2). CGCL is more common in females (3-6), with a female/male ratio of 1.3:1 (7). This lesion can occur at all ages, but most cases are diagnosed in the second and third decades of life (3,7). The mandible is usually more affected than the maxilla, with a mandible/maxilla incidence ratio of 2:1 (7). In radiographic analyses, CGCL may range from small apical lesions to large destructive multilocular radiolucencies involving large areas of the jaws (7).

Chuong et al. (2) first described aggressive and non-aggressive CGCL. The former is characterized by one or more of the following: pain, paresthesia, root resorption, rapid growth, cortical perforation, and a high recurrence rate. Non-aggressive lesions present with slower growth and without cortical perforation or tooth resorption. Aggressive lesions are usually larger and more frequently produce swelling (2). The pathogenesis of CGCL has yet to be elucidated.

Surgery is currently the most common proposed treatment for CGCL in the literature (5,6,8,9); surgical treatment methods range from simple curettage to aggressive en-bloc resection (4-6,10), which can lead to significant facial disfiguration. Intralesional corticosteroid injections are increasingly being used in the clinic, and some reports have shown excellent results. Intralesional corticosteroid injections can avoid extensive mutilating surgeries and successfully manage CGCL; the injections can be used alone or in combination with other treatment options, such as calcitonin or surgery (11). As most of the published articles on intralesional corticosteroid injections are case reports, the literature lacks data about this treatment modality. The aim of this study was to perform a meta-analytic study of intralesional corticosteroid injections for the treatment of CGCL.

## Material and Methods

The articles referenced in the bibliography were collected through a search of PubMed, using the following keywords: central giant cell granuloma, central giant cell lesion, and intralesional corticosteroid. Study articles and case report articles were selected. Case reports were included, as only one research article has been published on this topic. The time parameters of the search were set between 1988 and 2011. Additionally, the report by Terry and Jacoway (12) was included in this review, as this was the first report to document intralesional corticosteroid treatment for CGCL. The data were grouped into tables 1,2,3.

The inclusion criteria were as follows: articles published between the years 1988 and 2011 and cases using intralesional corticosteroid injections as the first choice for treatment of CGCL. The following exclusion criteria were used: studies that included reports on peripheral giant cell lesion and those that used a combination treatment of intralesional corticosteroids with other treatment methods, such as calcitonin or surgery, as the first therapeutic choice. Fourteen articles that met the inclusion criteria were selected. Of the articles selected, one was a research article, and thirteen were case reports.

The data obtained were analyzed for the following variables: number of cases, gender, mean age, location, aggressiveness of CGCL, drug and protocol used, whether any additional procedures were necessary, result of the treatment and follow-up. The aggressiveness of CGCL was defined as proposed by Chuong at al. (2) using data available from the articles. Non-aggressive lesions were those that presented as slow growing and without symptoms, cortical perforation, or root resorption. Aggressive lesions were those associated with pain, rapid growth, cortical perforation, root resorption, or a large size. Treatment results were analyzed as proposed by Nogueira et al. (13) using a four-item scoring system: A score of 1 indicated stabilization or regression in lesion size, as evaluated by the clinical aspect of the lesion and follow-up radiographs. A score of 2 represented the absence of symptoms. A score of 3 indicated an increased radio-opacity in the radiographs, representing peripheral and/or central calciﬁcation of the lesion. A score of 4 indicated an increasing difficulty in a solution diffusing into the lesion upon multiple applications. When a case was positive for all four scores, the response was classified as good, two or three scores as moderate, and one or zero scores implied a negative response to the treatment. If a case report did not indicate that the lesion was increasing in size or that the symptoms had not been controlled, these items were considered to have not happened, and the scores 1 and 2 were given to the report.

## Results

The search resulted in a total of 14 articles, with 13 case or series reports (12,14-25) and one research article (13). A sample of 41 patients was obtained (20 males and 21 females), with a female/male ratio of 1:0.95. The average age was 15.9 years; for aggressive lesions, the average age was 13.9 years, and for non-aggressive lesions, the average age was 18.3 years old. Twelve lesions were in the maxilla and 29 in the mandible, with a maxilla:mandible ratio of 1:2.4. According to the criteria defined by Chuong et al.(2) 23 lesions were classified as aggressive CGCL and 18 lesions as non-aggressive. Triamcinolone acetonide (10 mg/ml or 40 mg/ml) and triamcinolone hexacetonide (20 mg/ml) were the adopted drugs, but one case report did not indicate the type of corticosteroid used (20). The drugs were always diluted with an anesthetic solution of marcaine, lidocaine or bupivacaine in equal parts. A 2-ml dose of this solution for every 2 cm of radiolucency was the most cited dosage, but a dosage of 1 ml for every 1 cm3 was also reported. The most frequently used protocol was a regimen of 6 weekly injections, but a biweekly protocol was also described, and in one patient, 20 injections were given. According to the criteria previously defined by Nogueira et al. (13) in 2010, 32 (78.0%) cases were considered good results, 6 (14.6%) were considered moderate responses and 3 (7.3%) showed negative results to treatment (Data shown in  Table 1 Table 1 (continued), Table 2, Table 3).

**Table 1:**
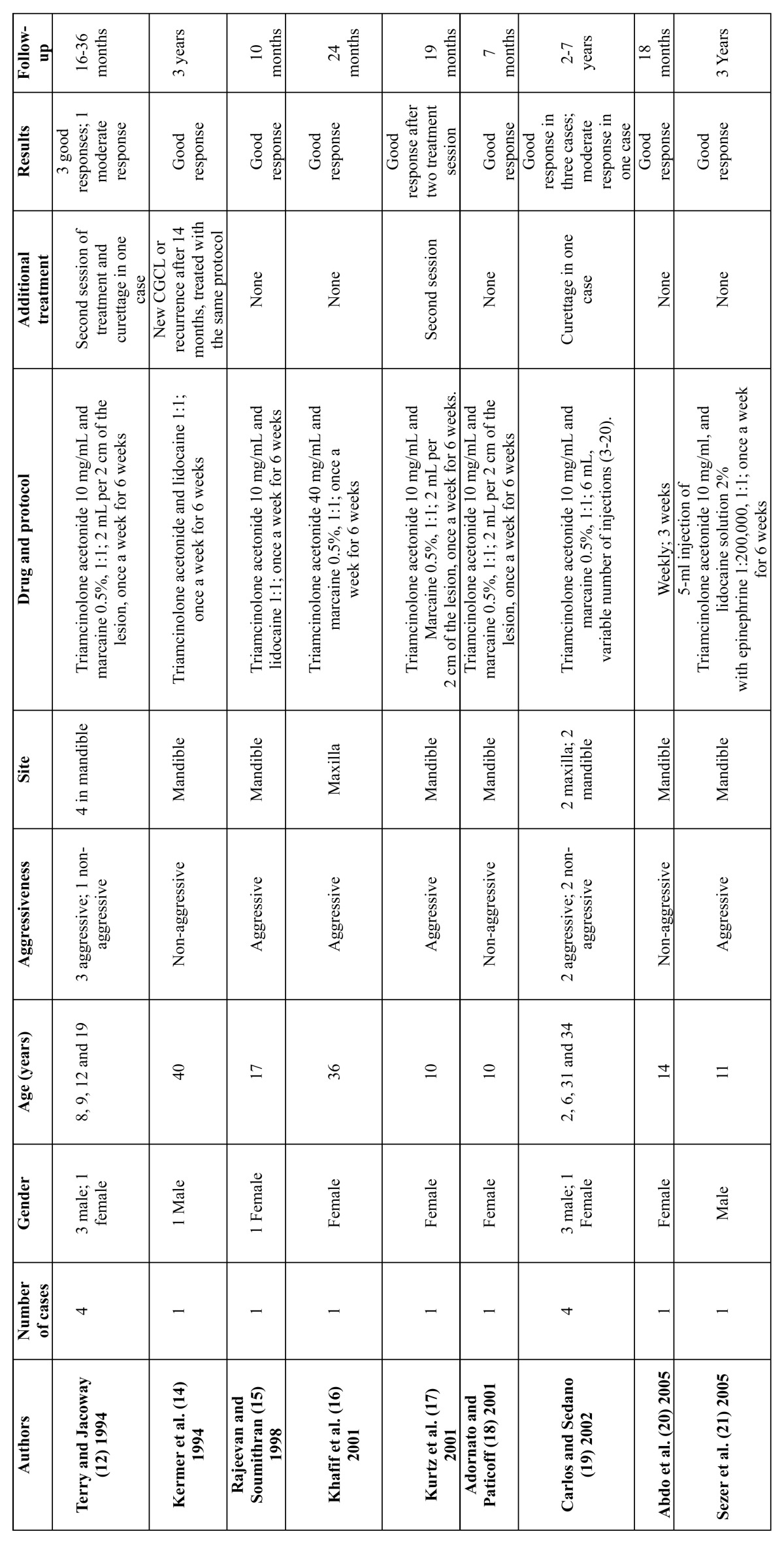
Characteristics of the central giant cell lesion studied.

**Table 1 (continued):**
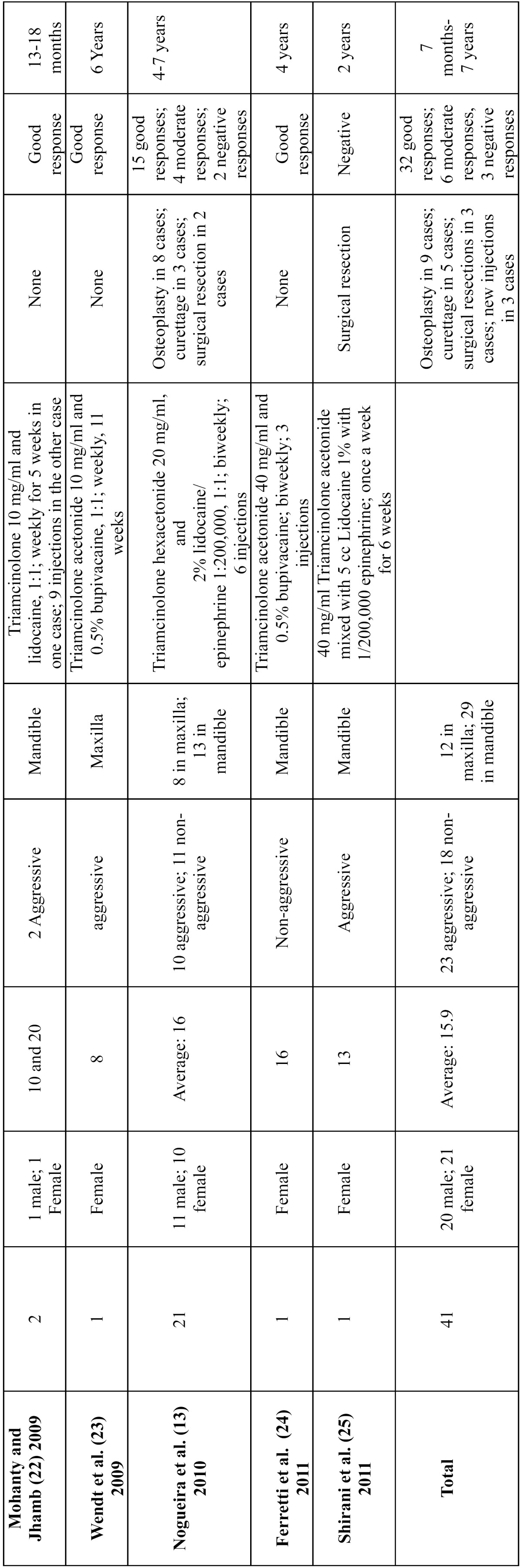
Characteristics of the central giant cell lesion studied.

**Table 2:**
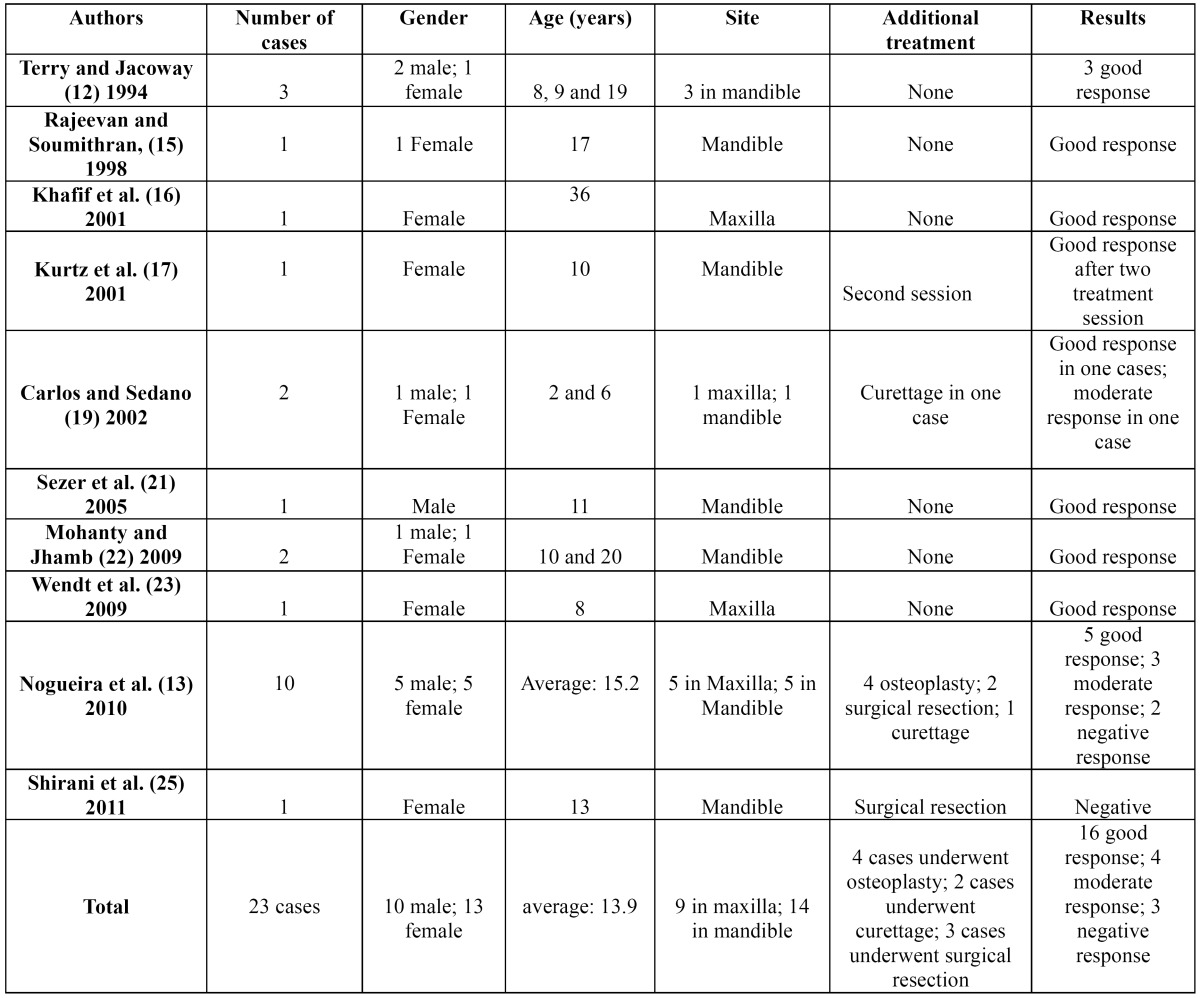
Characteristics of aggressive central giant cell lesion studied.

**Table 3:**
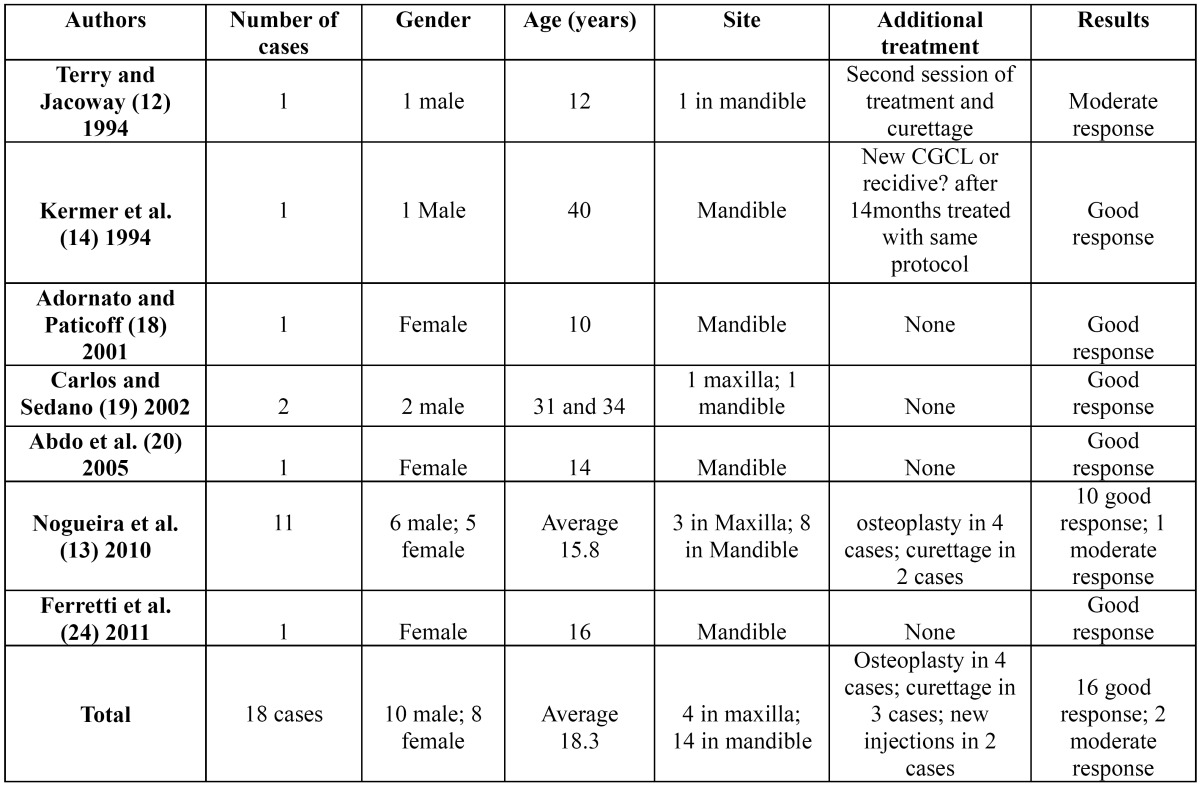
Characteristics of non-aggressive central giant cell lesions studied.

## Discussion

Surgery is the most common treatment of choice for CGCLs, and the extent of surgery ranges from curettage with or without adjuvant therapy, such as cryosurgery, peripheral ostectomy and carnoy solution, to aggressive en bloc resection (6,10), resulting in varying degrees of deformity (4). Conservative curettage and enucleation can lead to high recurrence rates, whereas en bloc resection may sacrifice adjacent structures, thus requiring surgical reconstructive procedures to recover satisfactory functional and esthetic results (2). As some cases of CGCL affect children and young adults, defects in the developing dentition and jaws are of great concern (13). Reconstructive and rehabilitative procedures, which usually require multiple hospitalizations, and the use of dental implants and prosthetic devices are very costly.

Several non-surgical methods have been proposed to treat CGCLs, including radiotherapy (26), systemic calcitonin (27-29), intralesional injection with corticosteroids (12-25) and systemic α interferon (30,31). Among these non-surgical treatment methods, intralesional corticosteroid injection has shown promising results and can lead to a complete resolution of the lesion or a significant reduction in size, allowing a more conservative surgery (12,13).

Body et al. (32) first reported the use of corticosteroids in the treatment of CGCL. In their report, the authors used dexamethasone to treat an aggressive recurrent case of CGCL, achieving a significant reduction in lesion size; however, complications resulted because a systemic corticosteroid was used. Consequently, the dose was reduced, and the lesion re-grew. Terry and Jacoway (12) first reported the use of intralesional corticosteroid injections in the treatment of CGCL. Intralesional injections are preferable because they can achieve an elevated and localized concentration in the tissue (12). In addition, the complications associated with systemic corticosteroid administration usually do not appear, as none of the articles included in this meta-analysis reported complications related to the corticosteroids.

The drugs used for treatment included triamcinolone acetonide (10 mg/ml or 40 mg/ml) and triamcinolone hexacetonide (20 mg/ml); both showed similar efficacies. The injections were administered weekly or biweekly. Nogueira et al. (13) proposed that using a more concentrated drug, such as triamcinolone hexacetonide 20 mg/ml, may allow for a biweekly interval, facilitating a greater reactivity and radiographic perception of the increasing radio-opacity. Six injections were the most common treatment regimen, but cases with up to 20 intralesional injections were reported (19). Triamcinolone is always diluted in equal parts with an anesthetic solution, and a dose of 2 ml for each 2 cm of radiolucency or 1 ml for each cm3 of lesion is injected.

Although CGCL is more frequently diagnosed in female patients (3-6), data from the present study found an almost equal frequency; 51.22% of cases occurred in female and 48.8% in male patients. Similar to other reports (3-7), in young patients, CGCL occurred more often in the mandible. In imaging studies, CGCL appeared as an uniloculated or multiloculated expansive osteolytic lesion and was frequently associated with tooth displacement (12,13,17,23-25). Root resorption was seen in some cases (12,13), as was cortical perforation (13,21,22,25); the later is better seen in computerized tomography (CT) scans. Microscopically, CGCL appeared as multinucleated giant cells in a cellular background composed of mononucleated stromal cells with an ovoid or spindle-shape, with hemorrhage foci (13,19,20,23,25). Two reports performed microscopic exams after the treatment and revealed a reduced number or, in some cases, the complete absence of CGCL, surrounded by markedly fibrocollagenous stroma and showing reduced vascularization (13,19).

According to the criteria defined by Chuong et al. (2), 23 lesions were classified as aggressive and 18 as non-aggressive. Aggressive lesions tended to affect younger patients compared with non-aggressive lesions, with an average age of 13.9 years for aggressive CGCL and 18.3 years for non-aggressive lesions. According to the criteria previously defined by Nogueira et al. (13), a good response to intralesional corticosteroid injections was seen in 78.0% of CGCL patients, and only 7.3% showed a negative result. Considering only aggressive lesions, 69.6% of cases presented a good response to treatment, and 13.0% showed a negative result. In non-aggressive CGCL, an even better result was found, as 88.9% these patients presented a good response to treatment, and none presented a negative result. These are excellent results, and it must not be forgotten that in cases showing a negative re-sponse to treatment, others treatment options are available. In fact, surgical resection was performed in all cases with a negative result (13,25). It has been previously described that CGCL contain glucocorticoid receptors in multinucleated giant cells and mononucleated stromal cells (33); this may be the reason why CGCL regresses upon corticosteroid treatment. More recently, Nogueira et al. (34) showed that in cases with good response to intralesional corticosteroid injections, a higher expression of glucocorticoid receptors were seen in multinucleated giant cells. Although in most cases intralesional injections were used alone, additional treatment may be necessary. Esthetic osteoplasty was performed in 9 cases in the present study. Curettage was performed in 5 cases, mainly to remove residual lesion tissue.

Altough the authors are aware of the limitations of meta-analytic studies, these data suggest that a non-surgical, intralesional corticosteroid injection approach for CGCL could be provided as a first-line treatment option. This treatment can lead to complete resolution of the lesion, or it may lead to a significant reduction in lesion size, allowing for a more conservative surgical approach. Further controlled prospective studies should be incentivized to corroborate these results.
